# A Protein's Role in Progressive Renal Disease

**DOI:** 10.1371/journal.pbio.0020194

**Published:** 2004-06-15

Focal segmental glomerulosclerosis (FSGS) made a brief media splash last year when a kidney transplant forced NBA superstar Alonso Mourning into early retirement. Mourning's condition elicited a flood of calls from fans offering their kidneys, but most people with kidney disease are not so lucky. Some 56,000 patients await transplants; many have waited over five years. FSGS, which underlies about 25% of the 60,000 kidney-related deaths each year, causes inflammation and irregular scarring in the glomeruli, clusters of blood vessels in the kidney that filter toxins from the blood. These lesions, which allow protein and blood to escape into the urine, cause progressive kidney failure. FSGS commonly occurs as an outgrowth of various primary disorders, including obesity, HIV infection, diabetes, and hypertension. Though it's not clear what causes FSGS, this form of renal pathology is becoming more common. By using the genes underlying inherited forms of FSGS as probes, scientists hope to uncover the mechanisms that unleash the disease and to find ways to stem the damage.[Fig pbio-0020194-g001]

**Figure pbio-0020194-g001:**
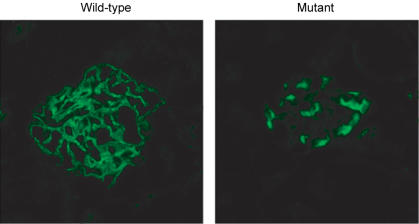
Mutant α-actinin-4 is mislocalizes and aggregates in renal glomeruli

Mutations in the *ACTN4* gene, which encodes a protein called α-actinin-4, cause an inherited form of FSGS. The protein normally remodels actin filaments, the primary structural component of muscle and cytoskeleton. Having a single mutated copy of the gene can cause FSGS in humans, though it is unclear how. In this issue of *PLoS Biology*, Martin Pollak and his colleagues at Brigham and Women's Hospital at Harvard Medical School use a three-pronged approach to figure out how the defective protein wreaks renal havoc and how these physiological changes lead to FSGS. Using biochemical analysis, cell-based studies, and a newly developed “knockin” mouse model, the researchers report that FSGS-related mutations cause α-actinin-4 to engage in various aberrant behaviors that ultimately rob the protein of its function and poison cells.

In previous experiments, Pollak's team had discovered that some families with the inherited form of FSGS carried mutations in *ACTN4*. In these individuals, the disease appeared to strike podocytes (glomeruli epithelial cells) first. While engineering mice designed to carry mutations in this gene, the researchers created mice that lacked detectable *Actn4* expression. These “knockout” mice developed severely damaged podocytes and progressive glomerular disease. In the current experiments, the researchers returned to their “knockin” mice, which carry two copies of the mutation found in the families with inherited FSGS. They also generated “normal” mice and mice harboring one normal and one mutant copy of the gene.

In the biochemical experiments, the researchers investigated the mutant protein's binding behavior. Typically, two α-actinin-4 proteins form a twosome without incident, but here the mutants behaved badly, assuming improper structural conformations and forming aggregates rather than pairs. Next, Pollak and colleagues introduced the genes with the *Actn4* mutation into podocytes, using a variety of methods, to see where in the cells the expressed proteins turned up. They also introduced fluorescently labeled mutant and normal *Actn4* genes into podocytes that were grown from the three mouse types: normal proteins were diffused throughout the cytoplasm in each cell type, but the mutant proteins showed an uneven distribution. Analysis of various tissues taken from the knockin mice revealed normal levels of mRNA transcripts—indicating normal gene transcription—but “markedly reduced” α-actinin-4 protein levels. The mutant proteins, it turns out, were manufactured normally but were degraded far more quickly than normal proteins. Electron microscopy showed that podocytes in the kidneys of the knockin mice had structural defects, while the mice themselves had significantly higher levels of protein in their urine than mice with one or two normal copies of the gene did.

The finding that FSGS-associated *Actn4* mutations produce α-actinin-4 aggregates with significantly reduced life span, the authors explain, suggests two possible mechanisms of initiating disease: aggregation and the toxic affects of aggregation could injure podocytes, or loss of α-actinin-4 function caused by rapid degradation of the protein could produce injury. Pollak and colleagues argue that both factors likely play a role: *Actn4* mutations lead to both reduced α-actinin-4 activity and protein aggregation, and the loss of protein function and the toxic effects of protein aggregation produce glomerular injury. As inherited renal disease typically emerges later in life, it may be that α-actinin-4 aggregation causes incremental but cumulative podocyte damage over time. So what does this mean for patients with progressive renal disease? While these findings may not translate into clinical applications anytime soon, they do suggest that therapies aimed at repairing the structure or expression of these essential cytoskeletal proteins might return the renegade proteins to the fold.

